# Exposure of honey bees to mixtures of microbial biopesticides and their effects on bee survival under laboratory conditions

**DOI:** 10.1007/s11356-024-32753-9

**Published:** 2024-03-07

**Authors:** Abdulrahim T. Alkassab, Silvio Erler, Michael Steinert, Jens Pistorius

**Affiliations:** 1 Institute for Bee Protection, Federal Research Centre for Cultivated Plants, Julius Kühn Institute (JKI), Messeweg 11-12, 38104 Braunschweig, Germany; 2 Zoological Institute, Technische Universität Braunschweig, Mendelssohnstraße 4, 38106 Brauschweig, Germany; 3 Institut Für Mikrobiologie, Technische Universität Braunschweig, Spielmannstraße 7, 38106 Braunschweig, Germany

**Keywords:** Biopesticides, *Apis mellifera*, *Bacillus*, *Beauveria*, Tank mixture, Survival

## Abstract

Biopesticides, having as active ingredients viruses, bacteria, or fungi, are developed to substitute or reduce the use of chemical plant protection products in different agrosystems. Though the application of mixtures containing several products is a common practice, interactions between microbial biopesticides and related effects on bees as non-target organisms have not been studied yet. In the current study, we exposed winter bees to five different microbial-based products and their combinations at the maximum recommended application rate to assess their responses. Laboratory oral exposure tests (acute/chronic) to single or binary products were conducted. Survival and food consumption of the tested bees were evaluated over the experimental duration. Our results show that some product combinations have potential additive or synergistic effects on bees, whereas others did not affect the bee’s survival compared to the control. Exposure of tested bees to the most critical combination of products containing *Bacillus thuringiensis aizawai* ABTS-1857 and *B. amyloliquefaciens* QST 713 strongly resulted in a median lifespan of 4.5 days compared to 8.0 and 8.5 days after exposure to the solo products, respectively. The exposure to inactivated microorganisms by autoclaving them did not differ from their respective uncontaminated negative controls, indicating effects on bee mortality might originate in the treatment with the different microorganisms or their metabolites. Further investigations should be conducted under field conditions to prove the magnitude of observed effects on bee colonies and other bee species.

**Supplementary Information:**

The online version contains supplementary material available at 10.1007/s11356-024-32753-9.

## Introduction

Recent reports have indicated increasing concerns regarding residue accumulation and potential adverse effects on the environment by chemical plant protection products (PPPs). Bees are exposed to residues of numerous active substances in various landscapes during their foraging activities (Böhme et al. 2018; Friedle et al. 2021; Mair and Wolf 2023). Among different stressors, exposure to pesticides at different concentrations in bee collected matrices, i.e. pollen and nectar, can cause complex responses at cellular mechanisms of the individuum, the whole organism, and colony/population level (e.g., Alkassab and Kirchner 2017; Siviter et al. 2021).

Nowadays, there is increasing interest in using sustainable biopesticides to reduce reliance on chemicals. Microbial biopesticides formulated from live microorganisms, including bacteria, fungi, and viruses, provide control options for several agricultural pests and diseases and fit well in integrated pest management (IPM) strategies (Quarles 2013). Such agents have been reported to have higher target-specificity than many synthetic chemicals (Mishra et al. 2020). However, their efficacy may be affected by various abiotic and biotic factors (Ownley et al. 1992). Various approaches have been suggested to overcome the limitations of efficacy, including the application of several products simultaneously by tank mixing to maximize efficacy and control a broad spectrum of pests in parallel (Bremmer et al. 2021; Haggag and Nofal 2006) and to overcome inconsistent performance under varying environmental conditions (Glare et al. 2012; Raymond et al. 2013; Van Lenteren 2012). Meshram et al. (2022) reported several ways of combinations, such as combining various microbes, combining different modes of action, and developing strain mixtures.

The simultaneous exposure, e.g., by tank mixing, to multiple microorganisms can lead to unpredictable effects on target or non-target organisms due to complex interactions. Some studies reported synergistic or antagonistic effects on target pests after combining microbial biopesticides (Garbutt et al. 2011; Hodgson et al. 2004; Li et al. 2021; Raymond et al. 2008). Soth et al. (2022) suggested that combining several genetically distinct isolates might lead to synergistic interactions or overcome environmental constraints. Studies using combinations of the two most applied entomopathogens, *Bacillus thuringiensis* and *Beauveria bassiana*, on target pests showed various interactions with different effects, including synergistic (Wraight and Ramos 2005; Kryukov et al. 2009; Xue et al. 2018), additive (Mwamburi et al. 2009), or antagonistic (Ma et al. 2008) depending on the pest and doses (Gao et al. 2012; Mantzoukas et al. 2013; Sayed and Behle 2017; Wraight and Ramos 2017). Nevertheless, combinations of microorganisms often provide more effective disease control than the single one (de Boer et al. 2003).

The use of biopesticides is expected to increase worldwide in the following years (Fenibo et al. 2021), and the simultaneous exposure of non-target organisms to multiple microorganisms should be considered and evaluated. Several guidelines have been developed to deal with the tank mixing of synthetic pesticides (Gandini et al. 2020), whereas similar guidelines are lacking for biopesticides. Some products are proven for compatibility with other products, and the label provides such information. However, this is not the case for the majority of microbial biopesticides.

Honey bees (*Apis mellifera*), as well as other pollinating insects, will be exposed to applied biopesticides by collecting, consuming, and storing contaminated pollen and nectar. In particular, honey bees are reported to perform hygienic behavior and grooming to minimize the risk of infections by fungi or bacteria (Facchini et al. 2019; Qu and Wang 2018). With respect to microbial biopesticides, Peng et al. (2020) reported that the average temperature of 33–36 °C within the colony during the season might work as a natural protection against fungal infections through fungal heat-inactivation. However, this cannot be generalized to all possible diseases caused by a fungal infection. Examples are fungal agents causing chalkbrood (*Ascosphaera apis*) and stonebrood (*Aspergillus flavus, A. fumigatus, A. niger*) in honey bee colonies. Despite an optimal temperature range of 30–35 °C for brood rearing, it is possible for *A. apis* to infect honey bee brood cells and cause chalkbrood (Flores et al. 1996).

Especially overwintering is a critical phase of colony development, where the colony forms a thermoregulating cluster to maintain the hive temperature between 24 and 34 °C during periods of cold temperatures (Heinrich 1981). Winter bees have a more extended survival capacity than summer bees. It is reported that winter bees have different physiology, e.g., a higher titer of yolk protein Vitellogenin, an enlarged fat body, and a higher number of hemocytes, which may lead to different sensitivity to infections by various microorganisms (Aurori et al. 2014; Remolina and Hughes 2008). While most studies analyzing microbial pesticides’ effects on honey bees work with summer bees (e.g., Malone et al. 1999; Renzi et al. 2016; Steinigeweg et al. 2021), the effects on winter bees are less investigated. To this end, the objective of the present study is to evaluate the effects of exposure to a tank mixture of several microbial biopesticides on long-living winter honey bees under laboratory conditions.

## Materials and methods

### Bee samples and experimental conditions

Winter worker honey bees (*Apis mellifera*) were collected from two healthy colonies maintained at the apiary of the Institute for Bee Protection of the Julius Kühn Institute in Braunschweig, Germany. All colonies had low levels of *Varroa* infections, where the natural mite falls lower than one per day. The treatment with formic acid against *Varroa* was conducted during summer 10 weeks before starting the experiment. Each colony had about 9000 workers and a fertile 1-year-old sister queen. Winter worker bees with undefined age were collected, shortly immobilized on ice and caged in groups of 10 bees. Each treatment group had four cages. The bees were incubated under controlled conditions (RH 65 ± 2%, 26 ± 2 °C, and darkness). For further details regarding cage description, see Alkassab and Kirchner (2016).

### Microbial biopesticides

Five microbial-based products, being commercially available in Germany, and their combinations at the maximum recommended application rate were tested (Table 1). Naturalis® contains 2.3 × 10^7^ CFU/ml of *Beauveria bassiana* (ATCC 74040), due to the available information on the product label. FlorBac® contains maximum 6 × 10^13^ CFU/kg of *Bacillus thuringiensis* spp. *aizawai* (ABTS-1857) (EFSA (European Food Safety Authority) 2020). Lepinox® Plus contains maximum 1.1 × 10^13^ CFU/kg *Bacillus thuringiensis* ssp. *kurstaki* (EG-2348) (EFSA (European Food Safety Authority) 2021a). Serenade® ASO contains maximum 3 × 10^13^ CFU/kg of *Bacillus amyloliquefaciens* (QST 713) (EFSA (European Food Safety Authority) 2021b). Madex® MAX contains nominal content of 3 × 10^13^ CpGV-M OB/L (EFSA (European Food Safety Authority) 2022). Biopesticides were prepared in the feeding solution (2 M sucrose *w/v*) at the manufacturer’s recommended concentrations. For combined treatments, both products were mixed at their individual recommended concentrations. The available information for compatibility with other products provided on the label was considered to select the combinations and mixing of different products.

**Table 1:** List of tested plant production products and their respective microorganisms as well as all tested combinations. The given maximum application rates according to the respective product labels were considered in the experiments to calculate the test concentrations

Product name	Microorganism	Maximum application rate	Tested concentrations	Tested combinations
FlorBac®	*Bacillus thuringiensis* ssp. *aizawai* (ABTS-1857)—*B.t.a*	1.5 kg/ha	1666 mg/l 10.0 × 10^10^ CFU/l	Naturalis® + FlorBac® FlorBac® + Serenade® ASO
Lepinox® Plus	*Bacillus thuringiensis* ssp. *kurstaki* (EG-2348)—*B.t.k*	1.0 kg/ha	2000 mg/l 2.2 × 10^10^ CFU/l	Naturalis® + Lepinox® Plus Lepinox® Plus + Madex® MAX
Serenade® ASO	*Bacillus amyloliquefaciens* (QST 713)—*B.a*	8.0 l/ha	40 ml/l 12.0 × 10^11^ CFU/l	Madex® MAX + Serenade® ASO FlorBac® + Serenade® ASO
Naturalis®	*Beauveria bassiana* (ATCC 74040)—*B. b*	2.0 l/ha	1.330 ml/l 3.1 × 10^7^ CFU/l	Naturalis ® + Madex® MAX Naturalis® + FlorBac® Naturalis® + Lepinox® Plus
Madex® MAX	*Cydia pomonella* granulovirus GV0006	150 ml/ha	0.125 ml/l 3.8 × 10^9^ CpGV-M OB/l	Lepinox® Plus + Madex® MAX Madex® MAX + Serenade® ASO

### Exposure protocols

Winter honey bees were exposed orally either acutely for 6–8 h (Organisation for Economic Co-operation and Development (OECD) 1998) or chronically over 10 days (Organisation for Economic Co-operation and Development (OECD) 2017) to the maximum recommended application rate of a single product or in a tank mixture of two microbial PPPs (Table 1). In the acute experiment, treated bees were starved for 90 min before providing 200 µl/cage of contaminated sucrose solution and were observed for 6–8 h. When this amount was consumed completely, the bees were fed ad libitum with sucrose solution (2 M) for the subsequent period. In the chronic experiment, treated bees were fed ad libitum with contaminated sucrose solution until day 10. For the subsequent 5 days, bees were fed with an uncontaminated sucrose solution (2 M). The control groups were fed ad libitum with an uncontaminated 2 M sucrose solution.

To evaluate the effect of adjuvants and co-formulants in the several products, an additional treatment group was exposed to inactivated microorganisms by autoclaving a stock solution (1% *w/w*) of the products solved in sterile water at 121 °C for 20 min. The *Bacillus thuringiensis (B.t.)*-based products were autoclaved twice to ensure the inactivation of *B.t*. spores. Thereafter, the autoclaved solutions were added at the same concentrations like the inactivated ones in sugar solution to evaluate their effects on bees. All test solutions were freshly prepared daily and fed to bees without storage to ensure high viability of all tested microorganisms. Mortality and food uptake were monitored daily for a total period of 15 days.

### Statistical analyses

To compare the different survival rates among groups over the test duration, Kaplan–Meier tests from the R package *survminer* were used (Kassambara et al. 2021). Bonferroni correction was used for multiple comparisons. Cox proportional hazard models from the R package *survival* (Therneau et al. 2020) were used to estimate hazard ratios (HR) for solo products and tank mixtures. The synergy index (*SI*_*AB*_) was calculated to determine the potential interaction between the tested combinations, applying the following formula designated by de Mutsert et al. (2009): *SI*_*AB*_ = (*HR*_*AB*_ − 1) / (*HR*_*A*_ + *HR*_*B*_ − 2). If the index was > 1, this is an indication of a synergistic effect, whereas an index *SI* = 1 or lower indicates no interaction effect. Linear mixed effects models (LMMs) were used to account for repeated measures and to test for differences in sucrose solution consumed per bee. Models were run with the function *lmer* from the *nlme* package (Pinheiro et al. 2021). All statistical analyses were conducted using R version 4.0.3 (R Core Team 2020) at a significance level of 0.05. Graphs were created using the *ggplot* function from the library *ggplot2* (Wickham et al. 2020).

## Results

### Chronic exposure

**Fig. 1:**
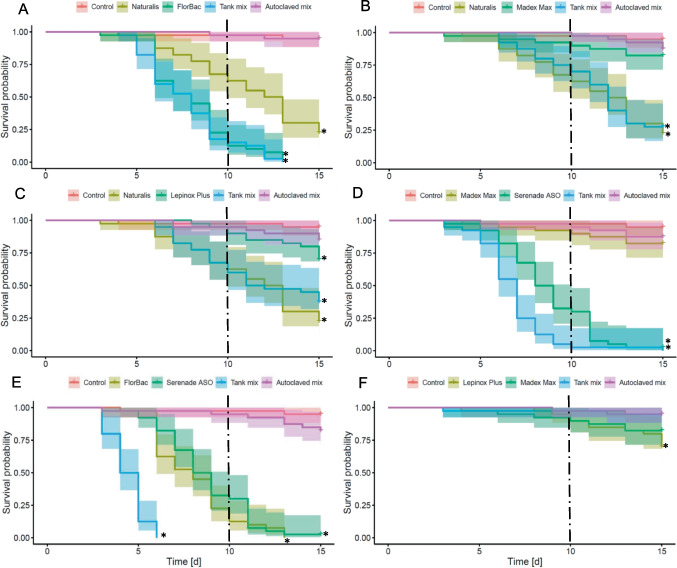
Survival rates of adult winter honey bees over 15 days after chronic exposure for 10 days (dashed lines) to different biopesticides (solo and mix), compared to autoclaved ones and the untreated control. **A** Naturalis®, FlorBac®, and their mixture; **B** Naturalis ®, Madex® MAX and their mixture; **C** Naturalis®, Lepinox® Plus, and their mixture; **D** Madex® MAX, Serenade® ASO and their mixture; **E** FlorBac®, Serenade® ASO and their mixture; **F** Madex® MAX, Lepinox® Plus, and their mixture. (*N* = 4 cages/treatment, *n* = 10 bees/cage; Kaplan–Meier tests; asterisk indicates *p* < 0.05 compared to control)

Comparing all effects of the different tank mixtures on honey bees, the results showed a strong variance in the observed effects. In the majority of tested mixtures, survival effects were driven mainly by the effects of the solo products (Naturalis® or FlorBac®), which caused the strongest effect (*p* < 0.001; Fig. 1A–C). Some mixtures indicated potential additive (Fig. 1D) or synergistic effects (Fig. 1E). Mixture of products containing *C. pomonella* GV0006 and *B.t.k.* EG-2348 did not affect the bee’s survival (*p* > 0.05; Fig. 1F).

The combination of products containing *C. pomonella* GV0006 and *B.a.* QST 713 caused significantly faster mortality by archiving the 50% mortality 36 h earlier than the solo product (*p* = 0.001; Fig. 1D). The median lifespan of the tested bees was 7.0 days after exposure to the mixture, compared to 8.5 and > 15 days after exposure to the products containing *B.a.* QST 713 or *C. pomonella* GV0006, respectively.

The critical mixture of products containing *B.t.a.* ABTS-1857 and *B.a.* QST 713 strongly reduced the survival time compared to the solo products (*p* < 0.001; Fig. 1E). The median lifespan of the tested bees was 4.5 days after exposure to the mixture, compared to 8.0 and 8.5 days after exposure to the products containing *B.t.a.* ABTS-1857 and *B.a.* QST 713, respectively. The autoclaved groups did not differ from their respective uncontaminated negative controls (*p* > 0.05; Fig. 1A–F).

### Acute exposure

**Fig. 2:**
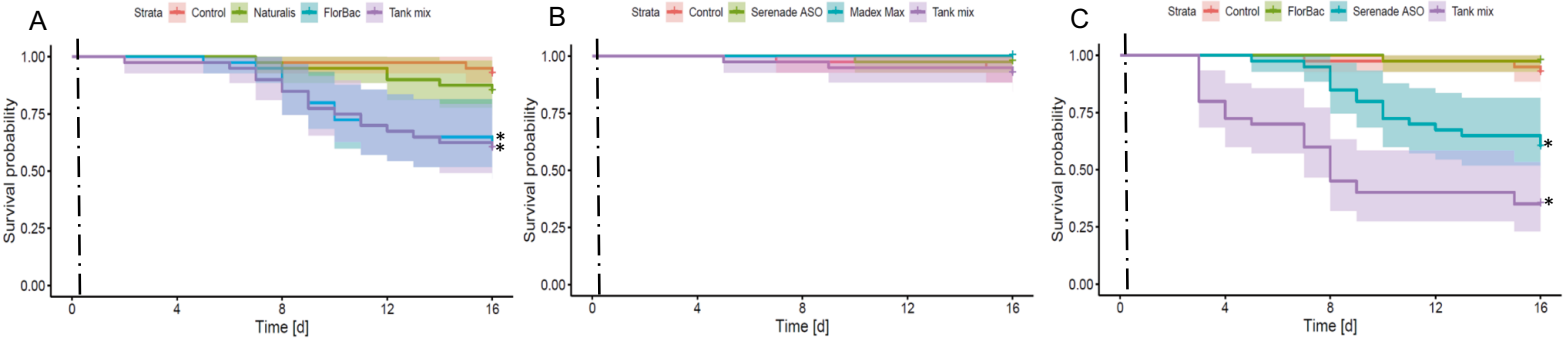
Survival rates of adult winter worker honey bees over 16 days after acute exposure for 6–8 h (dashed lines) to different biopesticides (solo and mix), compared to the untreated controls. **A** Naturalis®, FlorBac®, and their mixture; **B** Madex® MAX, Serenade® ASO and their mixture; **C** FlorBac®, Serenade® ASO and their mixture. (*N* = 4 cages/treatment, *n* = 10 bees/cage; Kaplan–Meier test; asterisk indicates *p* < 0.05 compared to control)

In general, acute exposure resulted in lower levels of mortality compared to chronic exposure to both solo products as well as their combinations (Fig. 1). The acute exposure to the mixture of products containing *B.t.a.* ABTS-1857 and *B.b.* ATCC 74040 caused an effect comparable to *B.t.a.* ABTS-1857 solo application (*p* < 0.001; Fig. 2A). Acute exposure to the mixture of products containing *B.t.a.* ABTS-1857 and *B.a.* QST 713 reduced the survival time significantly compared to the single products, which confirmed the results after chronic exposure but with lower mortality levels (*p* < 0.001; Fig. 2C). On the other hand, acute exposure to the mixture of products containing *B.a.* QST 713 and *C. pomonella* GV0006 did not affect the survival of tested bees (*p* > 0.05; Fig. 2B).

### Effects of tested combinations

Estimated hazard ratios (HR) show the probability of a living treated bee at a certain time point that will be dead by the next time point compared to an untreated bee (Fig. S1). These values were used to calculate the synergy index for each tank mixture. Two tank mixtures show potential synergism after chronic exposure (Table 2).

**Table 2:** Calculated synergy indices are based on the hazard ratio (HR) of each product after chronic or acute exposure to different tank mixtures. (*n.a.* not tested)

Tank mixtures	Synergy Index (SI)*	
	Chronic exposure	Acute exposure
**Naturalis® + FlorBac®**	0.86	0.86
**Naturalis® + Madex® MAX**	0.82	n.a
**Naturalis® + Lepinox® Plus**	0.61	n.a
**Madex® MAX + Serenade® ASO**	**2.16**	0.00
**FlorBac® + Serenade® ASO**	**7.50**	**3.27**
**Lepinox® Plus + Madex® MAX**	0.00	n.a

Bold emphasis number indicated the synergistic interactions between the products in the mixture a as SI > 1

*SI_*AB*_ = (*HR*_*AB*_ − 1) / (*HR*_*A*_ + *HR*_*B*_ − 2). *SI* > 1 indicates a synergistic effect, *SI* = 1 or lower indicates no interactions

The synergy indices were clearly above one for the tank mixture of the products containing *B.t.a.* ABTS-1857 and *B.a.* QST 713 after both acute (*SI* = 3.27) and chronic exposure (*SI* = 7.50), indicating a general synergism for this combination. The tank mixture of the products containing *B.a.* QST 713 and *C. pomonella* GV0006 showed potential synergism after chronic exposure (*SI* = 2.16) but not after acute exposure (*SI* = 0.00).

### Food consumption

**Fig. 3:**
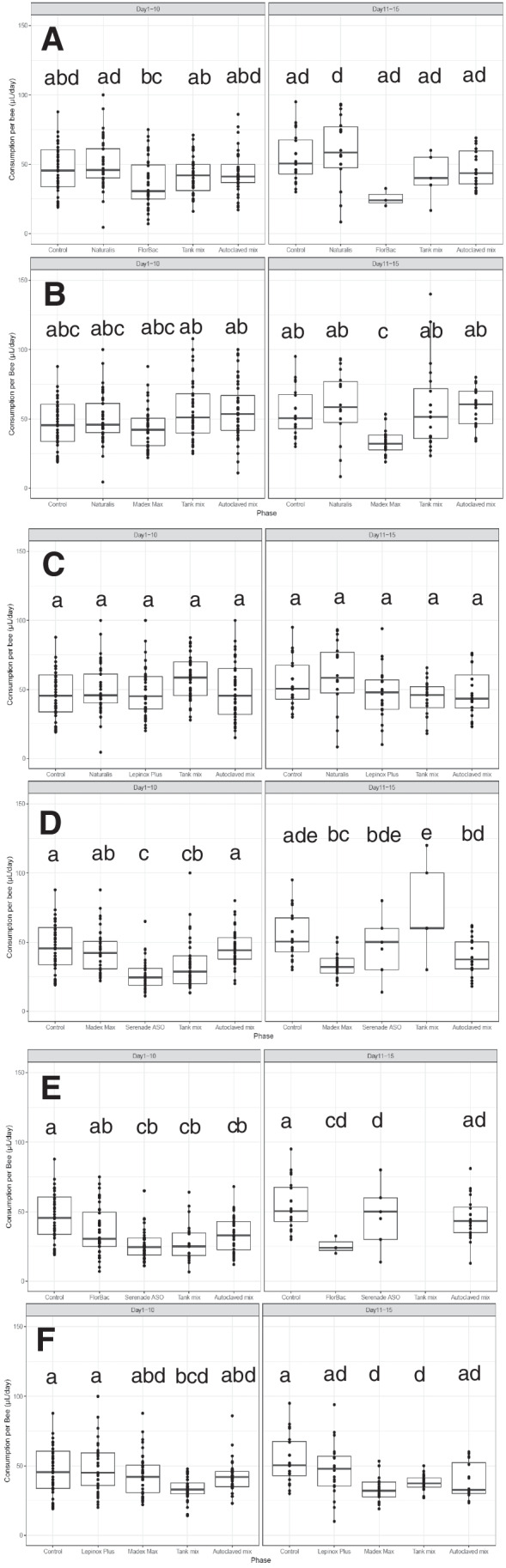
Daily food consumption (µl/bee/day) of chronically tested honey bees during exposure (days 1–10) and observation (days 11–15) phases. **A** Naturalis®, FlorBac®, and their mixture; **B** Naturalis ®, Madex® MAX and their mixture; **C** Naturalis®, Lepinox® Plus, and their mixture; **D** Madex® MAX, Serenade® ASO and their mixture; **E** FlorBac®, Serenade® ASO and their mixture; **F** Madex® MAX, Lepinox® Plus, and their mixture. Consumptions are shown as boxplots with median; the edges of the box indicate the 25th and 75th percentiles. Treatments not sharing the same letters indicate significant differences (*p* < 0.05)

Significantly lower consumption was observed during the chronic exposure phase to Serenade® ASO (26.8 ± 4.0 µl/bee) or tank mixtures containing this product, i.e., Serenade® ASO + Madex® MAX (33.6 ± 5.0 µl/bee) and Serenade® ASO + FlorBac® (27.6 ± 6.3 µl/bee), relative to the control (46.1 ± 5.7 µl/bee) (*p* < 0.05; Fig. 3D, E). Treated bees with tank mixture Lepinox® Plus + Madex® MAX consumed a significantly lower amount during exposure (33.7 ± 2.6 µl/bee) and post-exposure phase (37.6 ± 2.9 µl/bee) compared to control (46.1 ± 5.7 µl/bee in the first phase and 54.5 ± 2.9 µl/bee in the second phase) (*p* < 0.05; Fig. 3F). The exposed bees to Madex® MAX showed significantly lower consumption in the post-exposure phase (33.7 ± 4.4 µl/bee) relative to the negative control (*p* < 0.05; Fig. 3B, F). No effect on food consumption was observed for the autoclaved mixtures, neither during exposure nor post-exposure.

No significant differences between treatment groups were observed in food consumption after acute exposure, except for the autoclaved mix 1 and Florbac® (*p* > 0.05; Fig. S2).

## Discussion

Typically, the risk assessment for bees is conducted for each plant protection product before authorization (Krahner et al. 2022). Most commercial microbial biopesticides contain a single strain of a specific microorganism. Currently, several studies attempt to investigate the efficacy improvement by applying a combination of multiple microbial biopesticides based on the assumption that infections by more than one pathogen can synergistically affect suppressing pest populations (Garbutt et al. 2011; Wraight and Ramos 2005). However, a few synergistic interactions among biopesticides have been reported (Xu et al. 2011; Kryukov et al. 2009). In any case, non-target organisms, including pollinator insects, will be exposed to a mixture of applied microorganisms directly through the tank mixture or indirectly through the residues, i.e., the microorganisms or their metabolites. Recently, we reported that bees can be exposed chronically through stored pollen and nectar within the hive after application of a product containing *B.t.a.* in the field. This long-term presence of *B.t.a.* might be explained by differences between in-hive conditions compared to field conditions, where no more UV effects on the spores and/or the products within the hive are expected (Alkassab et al. 2022). Therefore, the evaluation of the combined effect has to include the potential effects on bees as non-target organisms.

Research investigating the effect of microbial biopesticides on honey bee health is still developing (Borges et al. 2021; Erler et al. 2022), particularly their interaction with the gut microbiome (Steinigeweg et al. 2023). In the present study, we reported for the first time how microbial combinations can affect honey bee survival under laboratory conditions. By observing no increased mortality for the autoclaved groups, it became apparent that the effects on bee mortality might originate in the treatment with the different microorganisms or their metabolites.

Our results show that a mixture of products containing *C. pomonella granulovirus* and *B. thuringiensis* ssp. *kustaki* did not affect the bee’s survival. This may be related to the high level of specificity of these microbials (Erler et al. 2022). However, slightly lower consumption during exposure phase was observed compared to the control. This may be related to different substances used in the formulated products. The observed effects of the other tested mixtures were driven mainly by the effects of the solo products, which caused the strongest effects. A critical mixture of products containing *B. thuringiensis* ssp. *aizawai* and *B. amyloliqueaciens* was identified, as the hazard ratio increased significantly compared with the solo products after acute and chronic exposure (Fig. S1).

The most studied combinations of microorganisms against target pests are *B.t.* and *B.b*. Lewis et al. (1996) reported enhanced suppression of the European corn borer after applying *B.t.* to *B.b.*-treated maize. Another study showed that applying a combination of *B.b.* and *B.t. tenebrionis* is more effective in controlling Colorado potato beetle larvae (Wraight and Ramos 2005). These effects may be related to the different modes of action of both microorganisms. *B.b.* can cause infections through the penetration of the cuticle, especially during the molting phase, and *B.t.* infects the digestive tract (Ma et al. 2008). Our results did not show such an increasing impact on adult winter honey bees after exposure to both microorganisms, as the tested bees were imagos, and thus, further investigations are still relevant to assess the effect of such combinations on bee larvae.

A strong combined effect of *B.t.* and *B. subtilis* was reported by Rajamanickam et al. (2015). They found that the lethal concentration values for the moth *Helicoverpa armigera* were significantly lower in the mixture than in *B.t.* and *B.s.* individually. This is in line with the results of this study, where the mixture reduced the lethal time compared to the solo products (Fig. 1). Although, *B. amyloliqueaciens* (formerly *subtilis*) is known to be effective against fungal plant diseases and produce several antibiotics as well as metabolites; among them, the metabolites surfactin and iturin have been reported to have insecticidal and insect antifeedant activity (Assié et al. 2002; Geetha and Manonmani 2010; Blibech et al. 2012). In the honey bee, the natural occurrence of several strains of *B. subtilis* was reported in the bee’s gut (Sabaté et al. 2009). On the other hand, ingestion of extrinsic strains of *B. subtilis* or its metabolites was shown to affect other insects adversely (Abd El-Salam et al. 2011; Ghribi et al. 2012). *B. thuringiensis* ssp. *aizawai* is an effective entomopathogen against lepidopteran larvae. However, its semi-specificity was indicated recently depending on the reported cross-effects across insect taxa and orders (e.g., Babin et al. 2020; Nawrot-Esposito et al. 2020; Steinigeweg et al. 2021, 2023; Tudoran et al. 2020; van Frankenhuyzen 2017). This shows that target-specificity is not always as strong as expected. Exposure to a mixture of spores with a different spectrum of metabolites from both bacterial species can increase the stress level within the digestive system. Potential digestive problems may be the result of a disruption in the microbiome’s function or of foreign microorganisms causing dysbiosis in the gut microbiome (Erler et al. 2022). A digestive problem may be related to the lower consumption rate during exposure to this mixture. Thus, more omics research can provide more information on the host’s physiology and metabolism related to the observed combined effects. Furthermore, the host species and inoculation strategy can affect the prevalence and consequences of co-infection situations. Studies investigating the interaction between microbials and their target and non-target host organisms will lead to a better understanding of their effects (Souza et al. 2019; Erler et al. 2022).

## Conclusion

The current study assessed separately the effects of five microbial biopesticides and some combinations to determine if there is a mixture with a critical impact on bees. Survival reductions were observed after chronic exposure to solo products containing *B.t.a. *ABTS-1857, *B.a.* QST 713, and *B.b.* ATCC 74040, whereas no effects of the products containing *C. pomonella* GV0006 and *B. t. k.* EG-2348 were detectable. Chronic exposure resulted in higher levels of mortality compared with acute exposure to solo products. This shows that combined exposure to microbial plant protection products has the potential, in some cases, to increase the risk for non-target organisms like honey bees. In such cases, further higher-tier studies with full-size colonies are needed to assess the response of honey bees and the related risk for honey bee colonies under field conditions.

Supplementary information.

### Supplementary Information

Below is the link to the electronic supplementary material.

Supplementary file1 (DOCX 341 KB)

## Data Availability

Data will be made available on reasonable request.

## Declarations

Ethics approval and consent to participate.

Not applicable.
