# Supplementary material for: Exposure of honey bees to mixtures of microbial biopesticides and their effects on bee survival under laboratory conditions
Source: Environ Sci Pollut Res Int. 2024 Mar 7;31(18):26618–27. doi: 10.1007/s11356-024-32753-9 (PMC11052877; doi:10.1007/s11356-024-32753-9)
Supplement: Supplementary file 1 — Supplementary file1 (DOCX 341 KB) [file 11356_2024_32753_MOESM1_ESM.docx]

**Supplementary materials**

**Fig. S1:** Hazard ratios (HR) of tested products and their combination after (A) chronic or (B) acute exposure. (K) control, (N) Naturalis^®^, (L.P) Lepinox^®^ Plus, (M.M) Madex^®^ MAX, (F.B); FlorBac^®^ (S.A.), Snerade ASO, (T1) Naturalis^®^ + FlorBac^®^, (T2) Naturalis^®^ + Madex^®^ MAX, (T3) Naturalis^®^ + Lepinox^®^ Plus, (T4) Madex^®^ MAX + Serenade^®^ ASO, (T5) FlorBac^®^ + Serenade^®^ ASO, (T6) Lepinox^®^ Plus + Madex^®^ MAX; Autoclaved mixtures (AT1- (*** p < 0.001, * p < 0.05). Vertical dashed lines represent a HR = 1. Each bar indicates HR and 95% confidence intervals.

**Fig. S2:** Daily food consumption of honey bees (µl/bee/day) after acute exposure. Treatments are shown as boxplots with median; the edges of the box indicate the 25^th^ and 75^th^ percentiles. Treatments not sharing the same letters indicate significant differences (*p* < 0.05).
